# Lower bounds for the low-rank matrix approximation

**DOI:** 10.1186/s13660-017-1564-z

**Published:** 2017-11-21

**Authors:** Jicheng Li, Zisheng Liu, Guo Li

**Affiliations:** 1 School of Mathematics and Statistics, Xi’an Jiaotong University, No. 28, Xianning West Road, Xi’an, 710049 China; 2 School of Statistics, Henan University of Economics and Law, No. 180, Jinshui East Road, Zhengzhou, 450046 China; 3 College of Mathematics and Statistics, Shenzhen University, No. 3688, Nanhai Ave, Shenzhen, 518060 China

**Keywords:** 15A23, 34D10, 68W25, 90C25, 90C59, low-rank matrix, approximation, error estimation, pseudo-inverse, matrix norms

## Abstract

Low-rank matrix recovery is an active topic drawing the attention of many researchers. It addresses the problem of approximating the observed data matrix by an unknown low-rank matrix. Suppose that *A* is a low-rank matrix approximation of *D*, where *D* and *A* are $m \times n$ matrices. Based on a useful decomposition of $D^{\dagger} - A^{\dagger}$, for the unitarily invariant norm $\|\cdot\|$, when $\|D\|\geq\|A\| $ and $\|D\|\leq\|A\|$, two sharp lower bounds of $D - A$ are derived respectively. The presented simulations and applications demonstrate our results when the approximation matrix *A* is low-rank and the perturbation matrix is sparse.

## Introduction

In mathematics, low-rank approximation is a minimization problem, in which the cost function measures the fit between a given matrix (the data) and an approximating matrix (the optimization variable), subject to a constraint that the approximating matrix has reduced rank. The problem is used for mathematical modeling and data compression. The rank constraint is related to a constraint on the complexity of a model that fits the data.

Low-rank approximation of a linear operator is ubiquitous in applied mathematics, scientific computing, numerical analysis, and a number of other areas. For example, a low-rank matrix could correspond to a low-degree statistical model for a random process (*e.g*., factor analysis), a low-order realization of a linear system [1], or a low-dimensional embedding of data in the Euclidean space [2], the image and computer vision [3–5], bioinformatics, background modeling and face recognition [6], latent semantic indexing [7, 8], machine learning [9–12] and control [13] *etc*. These data may have thousands or even billions of dimensions, and a large number of samples may have the same or similar structure. As we know, the important information lies in some low-dimensional subspace or low-dimensional manifold, but interfered with some perturbative components (sometimes interfered by the sparse component).

Let $D\in\mathbb{R}^{m\times n}$ be an observed data matrix which is combined as

1 $$ D = A + E, $$

where $A\in\mathbb{R}^{m\times n}$ is the low-rank component and $E\in \mathbb{R}^{m\times n}$ is the perturbation component of *D*. The singular value decomposition (SVD [14]) is a method for dealing with such high-dimensional data. If the matrix *E* is small, the classical principal components analysis (PCA [15–17]) can seek the best rank-*r* estimation of *A* by solving the following constrained optimization via SVD of *D* and then projecting the columns of *D* onto the subspace spanned by the *r* principal left singular vectors of *D*:

2 $$ \begin{aligned} &\min_{E} \|E \|_{F} \\ &\quad \text{s.t.}\quad\operatorname{Rank}(A) \leq r, \\ &\quad\phantom{\text{s.t.}} \quad\|D - A\|_{F}\leq\epsilon, \end{aligned} $$

where $r\ll\min(m,n)$ is the target dimension of the subspace, *ϵ* is an upper bound on the perturbative component $\|E\|_{F}$ and $\|\cdot\|_{F}$ is the Frobenius norm.

Despite its many advantages, the traditional PCA suffers from the fact that the estimation *Â* obtained by classical PCA can be arbitrarily far from the true *A*, when *E* is sufficiently sparse (relative to the rank of *A*). The reason for this poor performance is precisely that the traditional PCA makes sense for Gaussian noise and not for sparse noise. Recently, robust PCA (RPCA [6]) is a family of methods that aims to make PCA robust to large errors and outliers. That is, RPCA is an upgrade of PCA.

There are some reasons for the study of lower bound of a low-rank matrix approximation problem. Firstly, as far as we know, there is no literature to consider the lower bound of the low-rank matrix approximation problem. In our paper, we first put forward the lower bound. Secondly, for the low-rank approximation, when a perturbation *E* exists, there is an approximation error which cannot be avoided, that is, the approximation error cannot equal 0, but tends to 0. Thirdly, from our main results, we can clearly find the influence of the spectral norm ($\|\cdot\|_{2}$) on the low-rank matrix approximation. For example, for our main result of Case II, when the maximum eigenvalue of the matrix *D* is larger, the approximation error of $(D-A)$ is smaller. In addition, the lower bound can verify whether the solution obtained by algorithms is optimal. For details, please refer to the experiments Section 4 of our paper. Therefore, it is necessary and significant to study the lower bound of the low-rank matrix approximation problem.

### Remark 1.1

PCA and RPCA are methods for the low-rank approximation problem when perturbation item exists. Our aim is to prove that no matter what method is used, the lower bound of error always exists and it cannot be avoided with the perturbation item *E*. Considering the existence of error, this paper focuses on the specific situation of this lower bound.

### Notations

For a matrix $A\in\mathbb{R}^{m\times n}$, let $\|A\|_{2}$ and $\|A\| _{\ast}$ denote the spectral norm and the nuclear norm (*i.e*., the sum of its singular values), respectively. Let $\|\cdot\|$ be a unitarily invariant norm. The pseudo-inverse and the conjugate transpose of *A* are denoted by $A^{\dagger}$ and $A^{H}$, respectively. We consider the singular value decomposition (SVD) of a matrix *A* of rank *r*

$$A=USV^{H},\quad S=\operatorname{diag}\bigl(\{\sigma_{i}\}\bigr), 1\leq i\leq r, $$

where *U* and *V* are $m\times r$ and $n\times r$ matrices with orthonormal columns, respectively, and $\sigma_{i}$ is the positive singular values. We always assume that the SVD of a matrix is given in the reduced form above. Furthermore, $\langle A,B \rangle=\operatorname{trace}(A^{H} B)$ denotes the standard inner product, then the Frobenius norm is

$$\|A\|_{F}=\sqrt{\langle A,A \rangle}=\sqrt{\operatorname{tr}\bigl(A^{H} A \bigr)}= \Biggl(\sum_{i=1}^{m}\sum _{j=1}^{n} A_{ij}^{2} \Biggr)^{\frac{1}{2}}= \Biggl(\sum_{i=1}^{r} \sigma_{i}^{2} \Biggr)^{\frac{1}{2}}. $$

### Organization

In this paper, we study a perturbation theory for low-rank matrix approximation. When $\|D\|\geq\|A\| $ or $\|D\|\leq\|A\|$, two sharp lower bounds of $D - A$ are derived for a unitarily invariant norm respectively. This work is organized as follows. In Section 2, we provide a review of relevant linear algebra and some preliminary results. In Section 3, under different norms, two sharp lower bounds of $D - A$ are given for the low-rank approximation problem and some proofs of Theorem 3.5 are presented. In Section 4, example and applications are given to verify the provided lower bounds. Finally, we conclude the paper with a short discussion.

## Preliminaries

In order to prove our main results, we mention the following results for our further discussions.

### Unitarily invariant norm

An important property of a Euclidean space is that shapes and distance do not change under rotation. In particular, for any vector *x* and for any unitary matrices *U*, we have

$$\|Ux\|_{2}=\|x\|_{2}. $$

An analogous property is shared by the spectral and Frobenius norms: namely, for any unitary matrices *U* and *V*, the product $UAV^{H}$ is defined by

$$\big\| UAV^{H}\big\| _{p}=\|A\|_{p},\quad p=2, F. $$

These examples suggest the following definition.

#### Definition 2.1

([18])

A norm $\|\cdot\|$ on $\mathbb{C}^{m\times n}$ is unitarily invariant if it satisfies

$$\big\| UAV^{H}\big\| =\|A\| $$

for any unitary matrices *U* and *V*. It is normalized if

$$\|A\|=\|A\|_{2} $$

whenever *A* is of rank one.

#### Remark 2.2

Let $\Sigma= UAV^{H} $ be the singular value decomposition of the matrix *A* with order *n*. Let $\|\cdot\|$ be a unitarily invariant norm. Since *U* and *V* are unitary,

$$ \|A\| = \|\Sigma\|. $$

Thus $\|A\|$ is a function of the singular values of *A*.

The 2-norm plays a special role in the theory of unitarily invariant norms as the following theorem shows.

#### Theorem 2.3

([18])

*Let* $\|\cdot\|$ *be a family of unitarily invariant norm*. *Then*

3 $$ \|AB\|\leq\|A\|\|B\|_{2} $$

*and*

4 $$ \|AB\|\leq\|A\|_{2}\|B\|. $$

*Moreover*, *if* $\operatorname{Rank}(A)=1$, *then*

$$\|A\|=\|A\|_{2}. $$

We have observed that the spectral and Frobenius norms are unitarily invariant. However, not all norms are unitarily invariant as the following example shows.

#### Example 2.4

Let

$$ A=\left ( \textstyle\begin{array}{c@{\quad}c} 1 & 1 \\ 1 & 1 \end{array}\displaystyle \right ), $$

obviously, $\|A\|_{\infty} =2$, but for a unitary matrix

$$ U=\left ( \textstyle\begin{array}{c@{\quad}c} \frac{1}{\sqrt{2}} & \frac{1}{\sqrt{2}} \\ -\frac{1}{\sqrt{2}} & \frac{1}{\sqrt{2}} \end{array}\displaystyle \right ), $$

we have

$$ \|UA\|_{\infty} = \left\|\left ( \textstyle\begin{array}{c@{\quad}c} \frac{2}{\sqrt{2}} & \frac{2}{\sqrt{2}} \\ 0 & 0 \end{array}\displaystyle \right ) \right\|_{\infty} =\frac{4}{\sqrt{2}}. $$

#### Remark 2.5

It is easy to verify that the nuclear norm $\|\cdot\|_{\ast}$ is a unitarily invariant norm.

### Projection

Let $\mathbb{C}^{m}$ and $\mathbb{C}^{n}$ be *m* and *n*-dimensional inner product spaces over the complex field, respectively, and $A\in\mathbb{C}^{m\times n}$ be a linear transformation from $\mathbb{C}^{n}$ into $\mathbb{C}^{m}$.

#### Definition 2.6

([18])

The column space (range) of *A* is denoted by

5 $$ \mathcal{R}(A)=\bigl\{ x\in\mathbb{C}^{m}|x=Ay, y\in \mathbb{C}^{n}\bigr\} $$

and the null space of *A* by

6 $$ \mathcal{N}(A)=\bigl\{ y\in\mathbb{C}^{n}|Ay=0\bigr\} . $$

Further, we let ⊥ denote the orthogonal complement and get $\mathcal{R} = \mathcal{N}(A^{H})^{\perp}$ and $\mathcal{N}(A) = \mathcal {R}(A^{H})^{\perp}$.

The following properties [18] of the pseudo-inverse are easily established.

#### Theorem 2.7

([18])

*For any matrix* *A*, *the following hold*.

1. *If* $A\in\mathbb{C}^{m\times n}$ *has rank* *n*, *then* $A^{\dagger }=(A^{H} A)^{-1}A^{H}$ *and* $A^{\dagger}A=I^{(n)}$.
2. *If* $A\in\mathbb{C}^{m\times n}$ *has rank* *m*, *then* $A^{\dagger }=A^{H}(A A^{H} )^{-1}$ *and* $A A^{\dagger}=I^{(m)}$.

*Here* $I^{(n)}\in\mathbb{R}^{n\times n}$ *is the identity matrix*.

#### Theorem 2.8

([18])

*For any matrix* *A*, $P_{A} = AA^{\dagger}$ *is the orthogonal projector onto* $\mathcal{R}(A)$, $P_{A^{H}}= A^{\dagger}A$ *is the orthogonal projector onto* $\mathcal{R}(A^{H})$, $I - P_{A^{H}}$ *is the orthogonal projector onto* $\mathcal{N}(A)$.

### The decomposition of $D^{\dagger} - A^{\dagger}$

In this section, we focus on the decomposition of $D^{\dagger} - A^{\dagger}$ and a general bound of the perturbation theory for pseudo-inverses. Firstly, according to the orthogonal projection, we can deduce the following lemma.

#### Lemma 2.9

*For any matrix* *A*, $P_{A} = AA^{\dagger}$ *and* $P_{A^{H}}= A^{\dagger}A$, *then we have*

7 $$ P_{A}^{\bot}A=0,\qquad AP_{A^{H}}^{\bot}=0,\qquad P_{A^{H}}^{\bot}A^{H}=0,\qquad A^{\dagger}P_{A}^{\bot}=0. $$

#### Proof

Since $P_{A}^{\bot}= I-P_{A}$ and $P_{A^{H}}^{\bot}= I-P_{A^{H}}$, then we have that

$$ \begin{gathered} P_{A}^{\bot}A = (I-P_{A})A = A - A A^{\dagger}A = A - A A^{H} \bigl(A A^{H}\bigr)^{-1}A=0, \\ AP_{A^{H}}^{\bot} = A(I-P_{A^{H}})=A-AA^{\dagger}A=A-A \bigl(A^{H} A\bigr)^{-1}A^{H} A=0, \\ P_{A^{H}}^{\bot}A^{H} = A^{H} - P_{A^{H}}A^{H} = A^{H} - A^{\dagger}AA^{H} = A^{H} - \bigl(A^{H} A\bigr)^{-1}A^{H} A A^{H} = 0, \\ A^{\dagger}P_{A}^{\bot}= A^{\dagger}(I-P_{A})=A^{\dagger} - A^{\dagger}A A^{\dagger }=A^{\dagger} - \bigl(A^{H} A \bigr)^{-1}A^{H} A A^{\dagger}=0. \end{gathered} $$

The proof is completed. □

Using Lemma 2.9, the decompositions of $D^{\dagger} - A^{\dagger}$ are developed by Wedin [19].

#### Theorem 2.10

([19])

*Let* $D = A +E$, *then the difference* $D^{\dagger} - A^{\dagger}$ *is given by the expressions*

8 $$\begin{aligned} D^{\dagger}-A^{\dagger} =& -A^{\dagger}E D^{\dagger} - A^{\dagger}P_{D}^{\bot} + P_{A^{H}}^{\bot}D^{\dagger}, \end{aligned}$$

9 $$\begin{aligned} D^{\dagger}-A^{\dagger} =& -A^{\dagger}P_{A} E P_{D^{H}} D^{\dagger} - A^{\dagger}P_{A} P_{D}^{\bot} + P_{A^{H}}^{\bot}P_{D^{H}} D^{\dagger}, \end{aligned}$$

10 $$\begin{aligned} D^{\dagger}-A^{\dagger} =& -D^{\dagger}P_{D} E P_{A^{H}} A^{\dagger} + \bigl(D^{H} D\bigr)^{\dagger }P_{D^{H}}E^{H} P_{A}^{\bot} \\ &{}- P_{D^{H}}^{\bot} E P_{A}\bigl(AA^{H} \bigr)^{\dagger} . \end{aligned}$$

By Lemma 2.9, using $P_{A} = AA^{\dagger}$, $P_{A^{H}}= A^{\dagger}A$, $P_{A}^{\perp}=I-P_{A}$, $P_{A^{H}}^{\perp}=I-P_{A^{H}}$, these expressions can be verified.

In previous work [19], Wedin developed a general bound of the perturbation theory for pseudo-inverses. Theorem 2.11 is based on a useful decomposition of $D^{\dagger} - A^{\dagger}$, where *D* and *A* are $m \times n$ matrices. Sharp estimates of $\|D^{\dagger} - A^{\dagger}\|$ are derived for a unitarily invariant norm. In [20], Chen et al. presented some new perturbation bounds for the orthogonal projections $\|P_{D} - P_{A}\|$.

#### Theorem 2.11

([19])

*Suppose* $D = A + E$, *then the error of* $D^{\dagger} - A^{\dagger}$ *has the following bound*:

11 $$ \begin{aligned} \big\| D^{\dagger} - A^{\dagger}\big\| \leq \gamma\max\bigl\{ \big\| A^{\dagger}\big\| _{2}^{2}, \big\| D^{\dagger}\big\| _{2}^{2}\bigr\} \|E\|, \end{aligned} $$

*where* *γ* *is given in Table* 1.

**Table 1:** **Value options for** ***γ***

**∥⋅∥**	**Arbitrary**	**Spectral**	**Frobenius**
*γ*	3	$\frac{1+\sqrt{5}}{2}$	$\sqrt{2}$

#### Remark 2.12

For the spectral norm, by formula (11) we can achieve $\gamma= \frac {1+\sqrt{5}}{2}$. When $\|\cdot\|$ is the Frobenius norm, by formula (12), we have $\gamma= \sqrt{2}$. Similarly, for an arbitrary unitarily invariant norm, according to formula (13), we can deduce $\gamma=3$.

#### Remark 2.13

From Theorem 2.11, since $E= D-A$, in fact, if $\operatorname{Rank}(A)\leq \operatorname{Rank}(D)$, then (11) gives the lower bound of the low-rank matrix approximation:

12 $$ \begin{aligned} \|D - A\| \geq \frac{\|D^{\dagger} - A^{\dagger}\|}{\gamma\max\{\|A^{\dagger}\| _{2}^{2},\|D^{\dagger}\|_{2}^{2}\}}. \end{aligned} $$

In the following section, based on Theorem 2.11, we provide two lower error bounds of $D - A$ for a unitarily invariant norm.

## Our main results

In this section, we consider the lower bound theory for the low-rank matrix approximation based on a useful decomposition of $D^{\dagger} - A^{\dagger}$. When $\operatorname{Rank}(A)\leq \operatorname{Rank}(D)$, some sharp lower bounds of $D - A $ are derived in terms of a unitarily invariant norm. In order to prove our result, some lemmas are listed below.

### Lemma 3.1

([18])

*Let* $D = A + E$, *the projections* $P_{D}$ *and* $P_{A}$ *satisfy*

13 $$ P_{D} P_{A}^{\bot} = \bigl(D^{\dagger}\bigr)^{H} P_{D^{H}} E^{H} P_{A}^{\bot} = \bigl(P_{A}^{\bot} P_{D}\bigr)^{H}, $$

*therefore*

14 $$ \big\| P_{D} P_{A}^{\bot}\big\| \leq \big\| D^{\dagger}\big\| _{2} \|E\|. $$

*If* $\operatorname{Rank}(A)\leq \operatorname{Rank}(D)$, *then*

15 $$ \big\| P_{D}^{\bot}P_{A}\big\| \leq \big\| P_{D} P_{A}^{\bot}\big\| . $$

### Lemma 3.2

([21])

*Let* $A, D\in\mathbb{C}^{m\times n}$, $\operatorname{Rank}(A)=r$, $\operatorname{Rank}(D)=s$, $r\leq s$, *then there exists a unitary matrix* $Q\in\mathbb{C}^{m\times m}$ *such that*

16 $$ Q P_{A} Q^{H}=\left ( \textstyle\begin{array}{c@{\quad}c@{\quad}c} {I^{(r)}} & 0 & 0\\ 0 & 0 & 0\\ 0 & 0 & 0 \end{array}\displaystyle \right )\quad\textit{and}\quad Q P_{D} Q^{H}=\left ( \textstyle\begin{array}{c@{\quad}c@{\quad}c} \Gamma^{2}_{r} & 0 & {\Gamma_{r}}\Sigma_{r}\\ 0 & {I^{(s-r)}} & 0\\ \Sigma_{r}\Gamma_{r} & 0 & \Sigma_{r}^{2} \end{array}\displaystyle \right ), $$

*where*

$$ \Gamma_{r}=\left ( \textstyle\begin{array}{c@{\quad}c} \Gamma_{1} & 0 \\ 0 & I \end{array}\displaystyle \right ) \quad\textit{and}\quad \Sigma_{r}= \left ( \textstyle\begin{array}{c@{\quad}c} \Sigma_{1} & 0 \\ 0 & 0 \end{array}\displaystyle \right ) , $$

$\Gamma_{1}=\operatorname{diag} (\gamma_{1},\ldots, \gamma_{r_{1}})$, $0 \leq\gamma_{1} \leq \cdots\leq\gamma_{r_{1}}$ *and* $\Sigma_{1}=\operatorname{diag} (\sigma_{1},\ldots, \sigma_{r_{1}})$, $0 \leq\sigma_{1} \leq \cdots\leq\sigma_{r_{1}}$. *Moreover*, $\gamma_{i}$ *and* $\sigma_{i}$ *satisfy* $\gamma_{i}^{2} + \sigma_{i}^{2} =1$, $i=1, \ldots, r_{1}$.

According to Lemma 3.2, we can easily get the following result.

### Lemma 3.3

*Let* $A, D\in\mathbb{C}^{m\times n}$, $\operatorname{Rank}(A)=r$, $\operatorname{Rank}(D)=s$, $r\leq s$, *then we have*

17 $$ \big\| P_{A}^{\bot} P_{D}\big\| = \big\| P_{D} P_{A}^{\bot}\big\| . $$

### Proof

Since

18 $$ P_{A}^{\bot}=I - P_{A}= Q^{H} \left ( \textstyle\begin{array}{c@{\quad}c@{\quad}c} 0 & 0 & 0 \\ 0 & I^{(s-r)} & 0 \\ 0 & 0 & I^{(m-s)} \end{array}\displaystyle \right )Q, $$

and

19 $$ P_{D}=Q^{H} \left ( \textstyle\begin{array}{c@{\quad}c@{\quad}c} \Gamma^{2}_{r} & 0 & \Gamma_{r}\Sigma_{r}\\ 0 & I^{(s-r)} & 0\\ \Sigma_{r}\Gamma_{r} & 0 & \Sigma_{r}^{2} \end{array}\displaystyle \right )Q, $$

then

20 $$ P_{A}^{\bot} P_{D}=Q^{H} \left ( \textstyle\begin{array}{c@{\quad}c@{\quad}c} 0 & 0 & 0 \\ 0 & I^{(s-r)} & 0 \\ \Sigma_{r}\Gamma_{r} & 0 & \Sigma_{r}^{2} \end{array}\displaystyle \right )Q $$

and

21 $$ P_{D} P_{A}^{\bot}=Q^{H} \left ( \textstyle\begin{array}{c@{\quad}c@{\quad}c} 0 & 0 & \Gamma_{r}\Sigma_{r} \\ 0 & I^{(s-r)} & 0 \\ 0 & 0 & \Sigma_{r}^{2} \end{array}\displaystyle \right )Q. $$

Therefore, they have the same singular values which yield that $\|P_{A}^{\bot} P_{D}\| = \|P_{D} P_{A}^{\bot}\|$. □

This is a useful lemma that we will use in the proof of the main result. In order to prove our main theorem, two lower bounds of $D - A$ are required by the following lemma.

### Lemma 3.4

*For the unitarily invariant norm*, *if* $\operatorname{Rank}(A)\leq \operatorname{Rank}(D)$, *then the lower bound of* $D-A$ *satisfies*:

Case I: *For* $\| D\| \geq\|A\|$, *we have*

22 $$ \begin{aligned} \|D - A\| \geq\| D\| - \|A\| - \big\| D^{\dagger}-A^{\dagger}\big\| \|D\|_{2} \|A\|_{2}. \end{aligned} $$

Case II: *For* $\| D\| \leq\|A\|$, *we have*

23 $$ \begin{aligned} \|D - A\| \geq\|A\| - \|D\| - \big\| D^{\dagger}-A^{\dagger}\big\| \|D\|_{2}^{2}. \end{aligned} $$

### Proof

*Case I*: Since $\| D\| \geq\|A\|$, we have $\|D - A\| \geq \|D \| - \|A\|$. Using Theorem 2.3 and Lemma 3.1, we have $\|AB\|\leq\|A\| _{2}\|B\|$ and $\|P_{D}^{\bot}P_{A}\|\leq\|P_{D} P_{A}^{\bot}\|$, respectively. By Lemma 2.9, we have $P_{D}^{\bot}D=0$, $AP_{A^{H}}^{\bot}=0$ and $A^{\dagger}P_{A}^{\bot}=0$, this also yields

$$ \begin{aligned} \|D - A\| &\geq \|D \| - \|A\| = \|D\| - \big\| \bigl(P_{D} + P_{D}^{\perp}\bigr) \bigl(P_{A} + P_{A}^{\perp}\bigr) A \big\| \\ &= \|D\| - \big\| P_{D} P_{A} A + P_{D}^{\bot} P_{A} A\big\| \quad(\text{by Lemma 2.9}) \\ &= \|D\| - \|P_{D} P_{A} A \| -\big\| P_{D}^{\bot} P_{A} A\big\| \quad\bigl(\text{by } P_{D} \bot P_{D}^{\bot}\bigr) \\ &\geq\|D\| - \|A \| - \big\| P_{D}^{\bot} P_{A}\big\| \| A \|_{2} \quad(\text{by Lemma 3.1}) \\ &\geq\|D\| - \|A \| - \big\| P_{D} P_{A}^{\bot}\big\| \| A \|_{2} \\ &= \|D\| - \|A \| - \big\| D\bigl(D^{\dagger} - A^{\dagger}\bigr) P_{A}^{\bot}\big\| \| A\|_{2} \\ &\geq\|D\| - \|A \| - \big\| D^{\dagger} - A^{\dagger}\big\| \|D\|_{2} \| A\|_{2} \quad (\text{by Theorem 2.3}). \end{aligned} $$

*Case II*: Since $\| D\| \leq\|A\|$, we have $\|D - A\| \geq \| A \| - \|D\|$. Similarly, by Lemma 3.3, using $\|P_{A}^{\bot} P_{D}\| = \|P_{D} P_{A}^{\bot}\|$, we have

$$ \begin{aligned} \|D - A\| &\geq \|A \| - \|D\|= \|A\| - \big\| \bigl(P_{A} + P_{A}^{\perp}\bigr) \bigl(P_{D} + P_{D}^{\perp}\bigr) D\big\| \\ &= \|A\| - \big\| P_{A} P_{D} D + P_{A}^{\bot} P_{D} D\big\| \quad(\text{by Lemma 2.9}) \\ &= \|A\| - \|P_{A} P_{D} D \| -\big\| P_{A}^{\bot} P_{D} D\big\| \quad\bigl(\text{by } P_{A} \bot P_{A}^{\bot}\bigr) \\ &\geq\|A\| - \|D \| - \big\| P_{A}^{\bot} P_{D}\big\| \| D \|_{2} \\ &\geq\|A\| - \|D \| - \big\| P_{D} P_{A}^{\bot}\big\| \| D \|_{2} \quad (\text{by Lemma 3.3}) \\ &= \|A\| - \|D \| -\big\| D\bigl(D^{\dagger} - A^{\dagger}\bigr) P_{A}^{\bot}\big\| \| D\|_{2} \\ &\geq\|A\| - \|D \| -\big\| D^{\dagger} - A^{\dagger}\big\| \| D \|_{2}^{2}\quad (\text{by Theorem 2.3}). \end{aligned} $$

We complete the proof of Lemma 3.4. □

Our main results can be described as the following theorem.

### Theorem 3.5

*Suppose that* $D = A + E$, $\operatorname{Rank}(A)\leq \operatorname{Rank}(D)$, *for the unitarily invariant norm* $\|\cdot\|$, *the error of* $D - A$ *has the following bounds*.

Case I: *For* $\|D\|\geq\|A\|$, *we have*

24 $$ \begin{aligned} \|D-A\| \geq\frac{\| D\| - \|A\|}{1 + \gamma\max\{\|A^{\dagger}\|_{2}^{2},\| D^{\dagger}\|_{2}^{2}\}\|D\|_{2}\|A\|_{2}}. \end{aligned} $$

Case II: *For* $\|D\|\leq\|A\|$, *we have*

25 $$ \begin{aligned} \|D - A\| \geq\frac{\| A\| - \|D\|}{1 + \gamma\max\{\|A^{\dagger}\| _{2}^{2},\|D^{\dagger}\|_{2}^{2}\}\|D\|_{2}^{2}}, \end{aligned} $$

*where the value options for* *γ* *are the same as in Table* 1.

### Proof

*Case I*: For $\| D\| \geq\|A\|$, by Theorem 2.11 and Lemma 3.4 (22), we can deduce

$$ \begin{aligned} \| D\| - \|A\| &\leq\|D -A\| + \big\| D^{\dagger}-A^{\dagger} \big\| \|D\|_{2} \|A\|_{2} \\ &\leq\|D -A\| + \gamma\max\bigl\{ \big\| A^{\dagger}\big\| _{2}^{2}, \big\| D^{\dagger}\big\| _{2}^{2}\bigr\} \|D-A\| \|D\|_{2} \|A\|_{2} , \end{aligned} $$

this yields

26 $$ \begin{aligned} \|D-A\| \geq\frac{\|D\| - \|A\|}{1 + \gamma\max\{\|A^{\dagger}\|_{2}^{2},\| D^{\dagger}\|_{2}^{2}\}\|D\|_{2} \|A\|_{2} }. \end{aligned} $$

*Case II*: Similarly, for $\| D\| \leq\|A\|$, by Theorem 2.11 and Lemma 3.4 (23), we can deduce

$$ \begin{aligned} \| A\| - \|D\| &\leq\|D -A\| + \big\| D^{\dagger}-A^{\dagger} \big\| \|D\|_{2}^{2} \\ &\leq\|D -A\| + \gamma\max\bigl\{ \big\| A^{\dagger}\big\| _{2}^{2}, \big\| D^{\dagger}\big\| _{2}^{2}\bigr\} \|D-A\| \|D \|_{2}^{2} , \end{aligned} $$

this yields

27 $$ \begin{aligned} \|D-A\| \geq\frac{\|A\| - \|D\|}{1 + \gamma\max\{\|A^{\dagger}\|_{2}^{2},\| D^{\dagger}\|_{2}^{2}\}\|D\|_{2}^{2} }, \end{aligned} $$

where the value options for *γ* are the same as in Table 1. In summary, we prove the lower bounds of Theorem 3.5. □

### Remark 3.6

From the main theorem, we can see that if $\|D\|=\|A\|$, then $\|D - A \|=0$. However, in the problem of low-rank matrix approximation, $\|D\|$ is not necessarily equal to $\|A\|$, so the approximation error is present. Furthermore, when $\|D\|$ is close to $\|A\|$, simulations demonstrate that the error has a very small magnitude (see Section 4).

In this section, we discuss the error bounds under different conditions for the unitarily invariant norm. Based on a useful decomposition of $D^{\dagger} - A^{\dagger}$, for $\| D\| \geq\|A\|$ and $\| D\| \leq\|A\|$, we have bounds (26) and (27), respectively. The two error bounds are useful in low-rank matrix approximation. The following experiments illustrate our results when the approximation matrix *A* is low-rank and the perturbation matrix *E* is sparse.

## Experiments

### The singular value thresholding algorithm

Our results are obtained by a singular value thresholding (SVT [22]) algorithm. This algorithm is easy to implement and surprisingly effective both in terms of computational cost and storage requirement when the minimum nuclear norm solution is also the lowest-rank solution. The specific algorithm is described as follows.

For the low-rank matrix approximation problem which is contaminated with perturbation item *E*, we observe that the data matrix $D = A + E$. To approximate *D*, we can solve the convex optimization problem

28 $$ \begin{aligned} &\min\|A\|_{\ast} \\ &\quad\text{s.t.} \quad\|D - A\|_{F} \leq\varepsilon, \end{aligned} $$

where $\|\cdot\|_{\ast}$ denotes the nuclear norm of a matrix (*i.e*., the sum of its singular values).

For solving (28), we introduce the soft-thresholding operator $\mathcal{D}_{\tau}$ [22] which is defined as

$$\mathcal{D}_{\tau}(A):=U\mathcal{D}_{\tau}(S)V^{\ast},\quad \mathcal{D}_{\tau }(S)=\operatorname{diag}\bigl(\bigl\{ (\sigma_{i}-\tau)_{+}\bigr\} \bigr), $$

where $(\sigma_{i}-\tau)_{+}=\max{\{0,\sigma_{i}-\tau}\}$. In general, this operator can effectively shrink some singular values toward zero. The following theorem is with respect to the shrinkage operators [22–24], which will be used at each iteration of the proposed algorithms.

#### Theorem 4.1

([22])

*For each* $\tau> 0$ *and* $W\in\mathbb{R}^{m\times n}$, *the singular value shrinkage operator* $\mathcal{D}_{\tau}(\cdot)$ *obeys*

$$ \mathcal{D}_{\tau}(W)=\arg\min_{A} \tau\|A \|_{\ast} + \frac{1}{2} \|A - W\|_{F}^{2}, $$

*where* $\mathcal{D}_{\tau}(W):=U\mathcal{D}_{\tau}(S)V^{\ast}$.

By introducing a Lagrange multiplier *Y* to remove the inequality constraint, one has the augmented Lagrangian function of (28)

$$ \begin{aligned} L(A,Y)=\|A\|_{\ast} - \langle Y, A-D \rangle+\frac{\tau}{2}\|A - D\|_{F}^{2}. \end{aligned} $$

The iterative scheme of the classical augmented Lagrangian multipliers method is

29 $$ \left \{ \textstyle\begin{array}{l} A^{k+1}\in\arg\min_{A} L(A,Y^{k}),\\ Y^{k+1} = Y^{k} - \tau(D - A^{k+1}). \end{array}\displaystyle \right . $$

Based on the optimality conditions, (29) is equivalent to

30 $$ \left \{ \textstyle\begin{array}{l} \mathbf{0}\in\frac{1}{\tau}\partial(\|A^{k+1}\|_{\ast}) + A^{k+1} - (D + \frac{1}{\tau}Y^{k}), \\ Y^{k+1} = Y^{k} - \tau(D - A^{k+1}), \end{array}\displaystyle \right . $$

where $\partial(\cdot)$ denotes the subgradient operator of a convex function. Then, by Theorem 4.1 above, we have the iterative solution

31 $$ \left \{ \textstyle\begin{array}{ll} A^{k+1} = \mathcal{D}_{1/\tau}(D + \frac{1}{\tau}Y^{k}), \\ Y^{k+1} = Y^{k} - \tau(D - A^{k+1}). \end{array}\displaystyle \right . $$

The SVT approach works as described in Algorithm 1.

**Algorithm 1:**
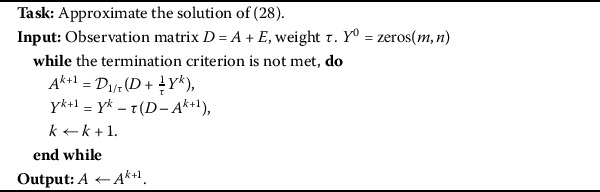
SVT

### Simulations

In this section, we use the SVT algorithm for the low-rank matrix approximation problem. Let $D = A + E\in\mathbb{R}^{m\times n}$ be the available data. Simply, we restrict our examples to square matrices ($m=n$). We draw *A* according to the independent random matrices and generate the perturbation matrix *E* to be sparse, which satisfies the i.i.d. Gaussian distribution. Specially, the rank of the matrix *A* and the sparse entries of the perturbation matrix *E* are selected to be $5\% m$ and $5\% m^{2}$, respectively.

Table 2 reports the results obtained by lower bounds (24), (25) and (12), respectively. Bounds (24) and (25) are our new result, bound (12) is the previous result. Then, comparing the bounds with each other by numerical experiments, we find that lower bounds (24), (25) are smaller than lower bound (12).

**Table 2:** **Lower bound comparison results**

	**Bound (** **24** **)**		**Bound (** **25** **)**		**Bound (** **12** **)**	
***m*** ** = ** ***n***	$\boldsymbol{\|\cdot\|_{2}}$	$\boldsymbol{\|\cdot\|_{F}}$	$\boldsymbol{\|\cdot\|_{2}}$	$\boldsymbol{\| \cdot\|_{F}}$	$\boldsymbol{\|\cdot\|_{2}}$	$\boldsymbol{\|\cdot\|_{F}}$
100	8.13e-7	1.89e-7	1.54e-7	3.31e-7	1.01e-4	1.27e-4
500	5.11e-8	3.71e-8	4.22e-8	4.62e-8	4.23e-4	5.22e-4
1,000	3.76e-8	2.14e-8	1.01e-8	1.19e-8	5.57e-4	7.48e-4

### Applications

**Figure 1:**
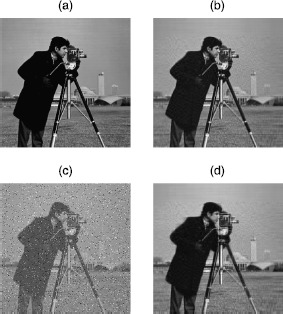
**Cameraman.** **(a)** Original $256\times256$ image with full rank. **(b)** Original image truncated to be rank 50. **(c)** 50% randomly masked of **(b)**. **(d)** Recovered image from **(c)**.

**Figure 2:**
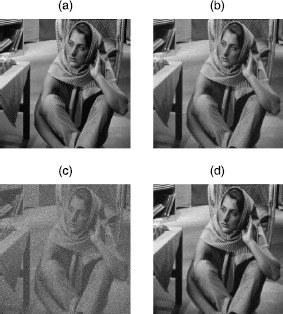
**Barbara.** **(a)** Original $512\times512$ image with full rank. **(b)** Original image truncated to be rank 100. **(c)** 50% randomly masked of **(b)**. **(d)** Recovered image from **(c)**.

In this section, we use the SVT algorithm for the low-rank image approximation. From Figures 1 and 2, comparing with the original image (a), the low-rank image (b) loses some details. We can hardly get any detailed information from incomplete image (c). However, the output image (d) $=A^{k}$, which is obtained by the SVT algorithm, can recover the details of the low-rank image (b). If we denote image (b) to be a low-rank matrix *A*, then image (c) is the observed data matrix *D* which is perturbed by a sparse matrix *E*, that is,

$$ \begin{aligned} \text{image (c) $=$ image (b)} + E. \end{aligned} $$

Using the SVT algorithm for the low-rank image approximation problem, the lower bound comparison results are shown in Table 3. We calculate $\|E\|_{F}=\|D - A \|_{F}$ are 8.71e-2 and 7.23e-2 for images Cameraman and Barbara, respectively. But for F-norm of our lower bound (25), we can see that they are 2.59e-5 and 1.09e-5 for images Cameraman and Barbara, respectively. That is to say, our error bounds can verify that the SVT algorithm still can be improved.

**Table 3:** **Lower bound comparison results of low-rank image approximation**

	**Cameraman**	**Barbara**
$\|E\|_{F}$	8.71e-2	7.23e-2
Bound (25)	2.59e-5	1.09e-5
Iters	200	200

## Conclusion

Low-rank matrix approximation problem is a field which arises in a number of applications in model selection, system identification, complexity theory, and optics. Based on a useful decomposition of $D^{\dagger} - A^{\dagger}$, this paper reviewed the previous work and provided two sharp lower bounds for the low-rank matrices recovery problem with a unitarily invariant norm.

From our main Theorem 3.5, we can see that if $\|D\|=\|A\|$, then $\|D - A \|=0$. However, in the problem of low-rank matrix approximation, $\|D\|$ is not necessarily equal to $\|A\|$, so the approximation error is present. Furthermore, from the main results, we can clearly find the influence of the spectral norm ($\|\cdot\|_{2}$) on the low-rank matrix approximation. For example, in Case II, when the maximum eigenvalue of the matrix *D* is larger, the error of $D-A$ is smaller.

Finally, we use the SVT algorithm for the low-rank matrix approximation problem. Table 2 shows that our lower bounds (24), (25) are smaller than lower bound (12). Simulation results demonstrate that the lower bounds have a very small magnitude. In applications section, we use the SVT algorithm for the low-rank image approximation problem, the lower bounds comparison results are shown in Table 3. From the comparison results, we find that our lower bounds can verify whether the SVT algorithm can be improved.
